# An Early Abdominal Wall Ectopic Pregnancy Successfully Treated with Ultrasound Guided Intralesional Methotrexate: A Case Report

**DOI:** 10.1155/2009/247452

**Published:** 2009-08-09

**Authors:** Paynesha M. Anderson, Erin K. Opfer, Jeanne M. Busch, Everett F. Magann

**Affiliations:** ^1^Department of Obstetrics and Gynecology, Naval Medical Center Portsmouth, Portsmouth, VA 23708, USA; ^2^Department of Radiology, Naval Medical Center Portsmouth, Portsmouth, VA 23708, USA

## Abstract

*Background*. The upper anterior abdominal wall is a very unusual location for an ectopic pregnancy making optimal management uncertain. *Case*. We report the case of a 26-year-old gravida 4, para 1, aborta 2 with a rising quantitative human chorionic gonadotropin level following a negative diagnostic laparoscopic examination. She was subsequently diagnosed with an abdominal wall ectopic pregnancy 2 cm inferior to the liver. A single percutaneous intralesional injection of methotrexate was successful after initial failure with systemic methotrexate. *Conclusion*. Systemic methotrexate is a logical first choice for management of a stable early abdominal wall pregnancy. Direct intralesional injection of methotrexate as the next treatment choice may avoid the morbidity linked with operative management.

## 1. Introduction

Abdominal ectopic pregnancies are extremely rare and account for only 1% of all ectopic pregnancies [1]. The gestational sac of an abdominal pregnancy usually implants in the pelvis or on highly vascular areas such as the liver, spleen, and mesentery [1] and can be associated with excessive maternal morbidity and mortality. The risk of maternal morbidity is 7-8 time greater with an abdominal ectopic pregnancy compared with other ectopic pregnancy locations and 90 times greater than an intrauterine pregnancy [2]. Given the rarity of an abdominal ectopic pregnancy and the potential mortality associated with abdominal pregnancies, early diagnosis and appropriate clinical treatment is essential. We present a unique case of failed systemic methotrexate therapy followed by a successful intralesional methotrexate injection for treatment of an infrahepatic abdominal wall ectopic pregnancy.

## 2. Patient History

A 26-year-old gravida 4, para 1, aborta 2 presented to an outside facility reporting spotting and cramping with a quantitative human chorionic gonadotropin (hCG) level of 8,979 mIU/mL. The patient was at 3 weeks and 2 days gestation by a sure last menstrual period. The patient had a Mirena IUD removed 2 days prior to her last menstrual period at her request because she desired to become pregnant again. The IUD at the time of removal was normally located. An ultrasound noted an empty uterus with an endometrial stripe of 7 mm and a complex cystic lesion of the left ovary which measured 6 × 3 × 5 cm. The patient was released to return the following day for a repeat quantitative hCG level which increased to 9,564 mIU/mL. She was then transferred to another outside facility for surgical management of a presumed ectopic pregnancy. Interval quantitative hCG level rose to 15,638 mIU/mL over the next 24 hours, and a diagnostic laparoscopy was performed. The complex appearing cystic left ovary was reported as benign in appearance and left in situ. There was no evidence of ectopic pregnancy observed. The upper abdomen was not reported as having been examined in the operative report.

Two days later the patient was referred to Naval Medical Center Portsmouth where she was admitted. Baseline serum laboratory values, tumor markers, and a repeat pelvic ultrasound were obtained. The ultrasound noted fluid in the posterior cul-de-sac, right ovary with a suspected corpus luteal cyst, left ovary with a conglomerate of hemorrhagic cysts, endometrial stripe of 3 mm, and no intrauterine pregnancy. Tumor markers were all normal, and the quantitative hCG level was reported as 23,274 mIU/mL. A D&C performed on the date of admission revealed no evidence of products of conception with a continued increasing quantitative hCG level on the following day to 25,678 mIU/mL.

**Figure 1:**
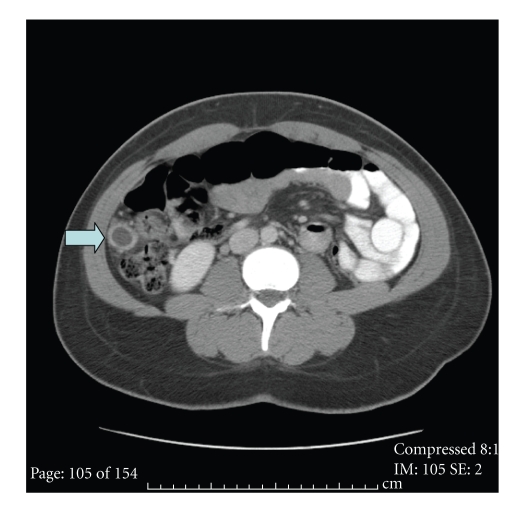
Computed tomography (CT) imaging of the abdomen revealing a 2.2 cm complex cystic structure 2 cm inferior to the inferior hepatic border which was suspicious for an ectopic abdominal gestational sac.

A CT scan of the chest, abdomen, and pelvis was performed to look for evidence of a possible hormone-secreting tumor or abdominal pregnancy causing the rising quantitative hCG levels. Computed tomography (CT) imaging of the abdomen revealed a 2.2 cm complex cystic structure 2 cm inferior to the inferior hepatic border which was suspicious for an ectopic abdominal gestational sac (Figure 1). A localized abdominal ultrasound was performed following the CT report; however, no specific ectopic could be identified by ultrasound.

**Figure 2:**
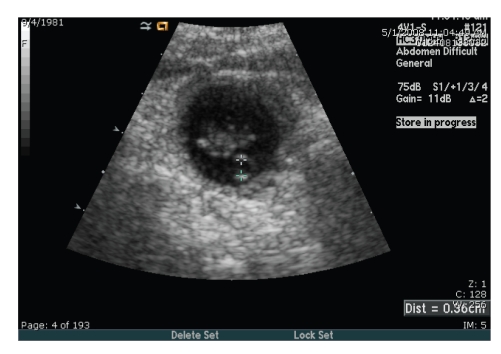
Initial ultrasound image of infrahepatic region of the abdominal wall noting a gestational sac with yolk sac and fetal pole.

The patient was counseled on treatment options including our recommendation of nonsurgical therapy with methotrexate. The planned therapy was 2 doses of methotrexate 50 mg/m^2^ IM on day 0 and day 4 as described by Barnhart to increase systemic methotrexate concentration with minimal overall toxicity. The quantitative hCG level on the day of treatment was 30,678 mIU/mL with a follow up quantitative hCG level on the fourth day following the initial treatment of 47,285 mIU/mL. Due to the 35% increase in quantitative hCG level following the first treatment, the 2-dose intramuscular plan was abandoned in favor of an intralesional injection planned for Day 7 in coordination with our Interventional Radiology Department given the patient's stable condition. The patient's quantitative hCG level slightly decreased to 44,637 mIU/mL by Day 7 when a formal ultrasound noted an ectopic abdominal pregnancy inferior to the liver with a crown rump length (CRL) consistent with an estimated gestational age of 7 weeks and 4 days. Fetal cardiac activity was demonstrated at 167 beats per minute (Figure 2).

**Figure 3:**
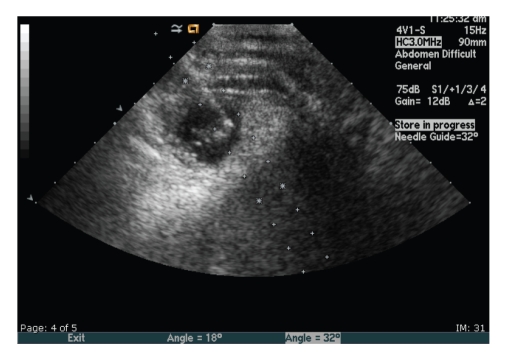
Intralesion injection of the gestational sac using a guided transducer.

The Interventional Radiology service was consulted and performed a direct injection of methotrexate into the gestational sac. A draped 4 MHz vector transducer with needle guide was placed transabdominally in the right upper quadrant to visualize the gestational sac. There was documented fetal cardiac activity prior to the procedure. The patient received conscious sedation with intravenous Versed and Fentanyl. A 10 cm, 20-gauge Chiba needle was then advanced into the gestational sac and appeared to be within the fetal pole (Figure 3). A total of 25 mg (0.25 mL) was introduced into the gestational sac. Immediate postprocedural ultrasonography demonstrated persistent cardiac activity of 156 beats per minute. There were no postprocedural complications.

An ultrasound examination performed the next morning documented continued fetal cardiac activity without significant interval change of the gestational sac. On day 4 after the intralesional methotrexate injection (Day 11 from the intramuscular injection of the first dose of methotrexate), the patient's quantitative hCG level had decreased to 35,622 mIU/mL. A repeat ultrasound examination at this time revealed absence of fetal cardiac activity.

The patient was discharged from the hospital and seen in follow up within 1 week. Her recovery following discharge from the hospital was uneventful, and she was returned to full activity by the third week after discharge. The only sequela noted from treatment was musculoskeletal pain at the site of injection. The patient's quantitative hCG levels were followed weekly until they were negative for 3 consecutive weeks. Fourteen weeks later the gestational sac remains clearly visible by bedside ultrasound, though the size has decreased to 1.6 cm, and the fetal pole and yolk sac are no longer visible.

## 3. Discussion

Abdominal ectopic pregnancies are rare and account for only 1% of all ectopic pregnancies [1]. Although these pregnancies are infrequently encountered, the possibility of an unusual location for an ectopic must always be part of the practitioner's differential diagnosis. It is extremely important to consistently perform a full visual survey of both the pelvis and upper abdomen during laparoscopy when evaluating for an ectopic pregnancy, especially if normal pelvic anatomy is noted, and this does not correlate with the observed quantitative hCG level. If clinical suspicion has not been confirmed by operative findings, radiographic scanning with CT or magnetic resonance imaging (MRI) may assist in diagnosis. Risk factors for an ectopic pregnancy should be considered additive and raise the index of suspicion with each additional risk factor possessed by the patient. This patient had a history of a Chlamydia infection and was also using an IUD for contraception. With calculation of the CRL of the fetus to date the gestational age of the pregnancy, we discovered that the patient had actually conceived prior to the removal of the IUD.

Although the diagnostic tools to identify an abdominal pregnancy are well established, the optimal treatment is less certain. To diagnose a primary abdominal pregnancy, the criteria described by Studdiford should be met: (1) normal tubes and ovaries, (2) no evidence of uteroperitoneal fistula, and (3) pregnancy related solely to the peritoneal surface and no evidence of secondary implantation following initial primary tubal nidation [1]. Because abdominal pregnancies typically implant on highly vascular surfaces such as the liver, spleen, omentum, large blood vessels, or abdominal serosa, the most minimally invasive but most effective means of treatment must be used.

Many different agents have been used to treat ectopic pregnancies including systemic and local methotrexate, local potassium chloride and hyperosmolar glucose, prostaglandins, danazol, etoposide, and mifepristone [3]. Most investigators have reported varying success rates in the medical treatment of abdominal pregnancies with local potassium chloride and/or local methotrexate, sometimes with the addition of systemic methotrexate [4]. When local treatment has failed, this has resulted in repeat dosing or surgical removal. Local injection has several advantages over systemic administration: increased local effectiveness with increased therapeutic levels of the drug at the site of the ectopic, decreased number of treatment courses, decreased systemic toxicity, and the ability to evaluate for the cessation of fetal cardiac activity.

There have been no reported cases in literature of the medical treatment of an abdominal ectopic implantation to the less vascular anterior abdominal wall unassociated with a prior low transverse abdominal wall scar. Given the close proximity to such a highly vascular organ, we sought to find the most minimally invasive treatment for our patient. Few case reports have observed success with only the intralesional injection of ectopic pregnancies in various locations such as the cervix, fallopian tube, or liver parenchyma [5] without the concomitant use of potassium chloride injection. Successful intralesional injection of methotrexate alone in an abdominal ectopic has only been reported in one case of an intrahepatic ectopic [5]. In our case, we observed a Day-4 increase of the quantitative hCG level by 35% following systemic methotrexate and determined that this was a poor prognostic factor for a second dose of systemic methotrexate. Our management was likely successful with a single dose of intralesional methotrexate because of the early gestational age. Avoidance of surgical removal of the ectopic lesion was accomplished with no long term sequelae exhibited by the patient. In the stable patient who fails systemic methotrexate therapy, intralesional injection of an abdominal wall ectopic pregnancy presents an effective, minimally invasive treatment option which should be attempted prior to surgical removal.
